# Caregiver support in aging societies: a qualitative metasynthesis informing public health policy

**DOI:** 10.3389/fpubh.2026.1821540

**Published:** 2026-06-11

**Authors:** Arielle Galinsky, Clara Sorkin, Elizabeth Marfeo

**Affiliations:** 1 Tufts University, Medford, MA, United States; 2 Yale Law School, New Haven, MA, United States; 3 Harvard University John F Kennedy School of Government, Cambridge, MA, United States

**Keywords:** caregivers, community based participatory research, dementia, health policy, public health, qualitative studies

## Abstract

**Objective:**

To examine and synthesize qualitative evidence on the experiences of older community-based caregivers of people living with dementia, guiding public health initiatives that address caregiving needs as a central priority in aging societies.

**Methods:**

Systematic search using online databases (Medline, PsycINFO, Google Scholar) for peer-reviewed literature published in the last 10 years. We used an iterative approach to extract qualitative data, perform thematic analysis, and conduct quality appraisal using CASP criteria. To translate synthesized insights into policy recommendations, we embedded this study within a community-based case study, co-designed with local stakeholders.

**Results:**

845 articles were identified for screening with 24 full text articles included in the analytic sample. Three key themes related to structural barriers, emotional impact, and caregiver identity were identified describing the multifaceted impact of caregiving on caregivers' lives.

**Conclusions:**

Caregiving experiences are shaped by complex structural limitations. Public health systems must recognize caregivers as essential to the healthcare continuum and invest in community-based support sustaining their capacity.

## Introduction

### Caregiving as a public health priority

As of 2025, in the United States, there are an estimated 63 million family caregivers who provide care to an older adult or child with a disability on an ongoing basis (1). This is a significant increase of nearly 45% from 2015. Family caregivers adopt a myriad of responsibilities to care for their loved one, from assisting with activities of daily living (ADLs) like bathing or dressing to supporting with financial decision making and end-of-life wishes (2). The number of individuals assuming the role of a family or “informal” caregiver in the US has significantly risen over the past decade. In 2020, more than 1 in 5 Americans identified as being an informal caregiver (3). In this paper, ‘caregiver' refers to an informal, or unpaid, family member (for example, a spouse, child, partner, friend, or neighbor) who delivers consistent assistance with ADLs and supports complex medical and coordination tasks. Importantly, an informal caregiver has a personal relationship with the individual, whereas a formal caregiver, who is financially compensated to deliver services, might not.

While research indicates that caregiving can provide positive outcomes such as feelings of meaning and purposefulness, the burden of caregiving has also been documented as becoming increasingly difficult in recent years (4). In a 2020 report, approximately 23% of caregivers attributed their caregiving role as making their own health worse, 45% indicated a significant financial impact of caregiving, and many reported difficulties in coordinating care across complex providers and health systems in the past 5 years (3).

Whether positive or negative, the evidence is clear that caregiving is associated with important quality of life outcomes for both caregivers and their care partner (5). Furthermore, the identity of a caregiver is hardly universal. Informal caregivers may vary in the way in which they conceptualize their caregiving role based on a variety of factors such as their relationship with the care partner, degree of choice in caregiving, and nature of health condition with which their care partner is living (6). Important gaps in existing research remain regarding (1) how recent shifts in existing US health systems and structures may have altered trends observed in the past, (2) the degree to which evidence fully represents a diversity in caregiver perspectives, and (3) development of innovative community-based solutions to support caregivers as the US population ages.

### Caregiver burden for individuals with dementia

In comparison with other chronic conditions, family caregivers for an individual with dementia, often termed as “invisible second patients,” involves extensive supervision, oversight of both behavioral and psychological systems (7). This role can produce cumulative stressors to the individual providing care, with adverse effects including social isolation and depression (8). The economic value of this informal care is estimated at nearly $413 billion, (1) underscoring the significant societal reliance on family caregivers. From a public health perspective, this highlights an urgent need for supportive interventions, accessible mental health services, respite programs, and policies aimed at reducing caregiver burden and preventing long-term health consequences.

### Existing public health resources for family caregivers

Although a wide range of supports is available to family caregivers, access is highly variable and influenced by socio-economic and geographic factors, contributing to notable gaps and disparities in service utilization (9). Social-based offerings often include support groups among caregivers, which have both an online and in-person presence in many communities. Disease specific associations, like the American Cancer Society and the American Parkinson Disease Association, also offer their own opportunities for connection among patient and caregivers alike. Local Areas on Aging might be able to connect family caregivers with local organizations that offer occasional services for chore, maintenance, or yard help (10). In some states, family caregivers might have the opportunity to receive a financial stipend for their work. Some states have what is known as Consumer-Directed Person Assistance Programs (or CDPAP) that provides financial support to family caregivers, with variability of resources and amount among each state. Some states also offer tax credits to caregivers for up to a few thousand dollars every year, as well as respite offerings to cover care. For example, in 2024, Oklahoma implemented the Caring for Caregivers Act, which provides Oklahomans with $2,000–$3,000 in tax credits for eligible expenses (11). In Massachusetts, the state's Family Caregiver Support Program offers, at no cost to the caregiver recipient, direct counseling services, and respite care. Furthermore, recognizing the economic ramifications of caregiving, the private sector has also begun to step up to meeting care needs (12). Over the past decade, it has become more of a widespread practice for employers to offer caregiving education benefits and digital tools to help families navigate caregiving such as HomeThrive™ (13) and Voya^®^ (14).

However, barriers to these resources remain persistent, particularly among older caregivers who are not eligible for employment-related caregiver benefits programs. This paper describes a Community-Based Participatory Research (CBPR)-informed systematic review and metasynthesis examining persistent gaps in support for older family caregivers, particularly those caring for individuals with Alzheimer's disease and related dementias (ADRD). By using a qualitative synthesis approach, this paper aims to integrate findings and highlight actionable, context-sensitive barriers that generalize more widely than single-site studies. Clarifying which gaps are consistent, and where they remain, is critical so policy and practice can effectively produce solutions. Furthermore, this study integrates Community-Based Participatory Research (CBPR) principles (15) to ensure that the research process is collaboratively grounded and oriented toward the development of actionable recommendations. These recommendations are designed to be directly relevant to both caregivers and community-based organizations that provide programs and resources for older adults.

## Methods

This systematic review and metasynthesis follows the protocol submitted and registered on PROSPERO (registration number CRD42024546902). All components of this study that involved CBPR methods were approved by the local university IRB review board.

### Research design

This study used an integrative community-based qualitative approach combining a systematic review, meta-synthesis, and a community-based case study. Qualitative data was used to perform a meta-synthesis to integrate first- and second-order findings from included studies and to generate higher-order themes relevant to implications for advancing health policy, community-based programs, and services for older caregivers of people living with dementia and related disorders. To translate synthesized insights into practice, we embedded this study within the context of a collaborative community-based case study, co-designed with local stakeholders (16).

### Review search strategy

A comprehensive literature search was conducted in PubMed, MEDLINE, CINAHL, and Google Scholar to identify qualitative studies on caregiving experiences among older adults providing care in community-based settings. Studies were included if they reported qualitative findings on the experiences, daily life, or support needs of family or informal caregivers of older adults (65+) with dementia or related cognitive impairment living in a US. context. Articles had to be full-text, English-language, including primary data from research using qualitative methods. Studies were excluded if they focused on clinical interventions, were not qualitative or if mixed methods had insufficient qualitative data reported, involved care recipients residing in residential care settings, or included a comparative international view

The initial PubMed search string was:

((“qualitative”[All Fields] OR “qualitatively”[All Fields] OR “qualitative”[All Fields]) AND (“caregiver s”[All Fields] OR “caregivers”[MeSH])
Terms] OR “caregivers”[All Fields] OR “caregiver”[All Fields] OR “caregiving”[All Fields])) AND ((y_10[Filter]) AND (fft[Filter]) AND (80 and over[Filter] OR aged[Filter]))

This search, with filters applied for full-text availability, publication within the last 10 years, and study participants aged ≥65 years (including ≥80 years), yielded n = 3,255 studies. Restricting to English-language publications reduced the yield to n = 3,179. The search was then refined to focus on dementia-related caregiving using the terms:

(qualitative caregivers AND ((y_10[Filter]) AND (fft [Filter]) AND (English [Filter]) AND (aged [Filter] OR 80andover [Filter]))) AND (dementia OR MCI OR Alzheimer's OR ADRD OR “cognitive impairment”)

**Figure 1:**
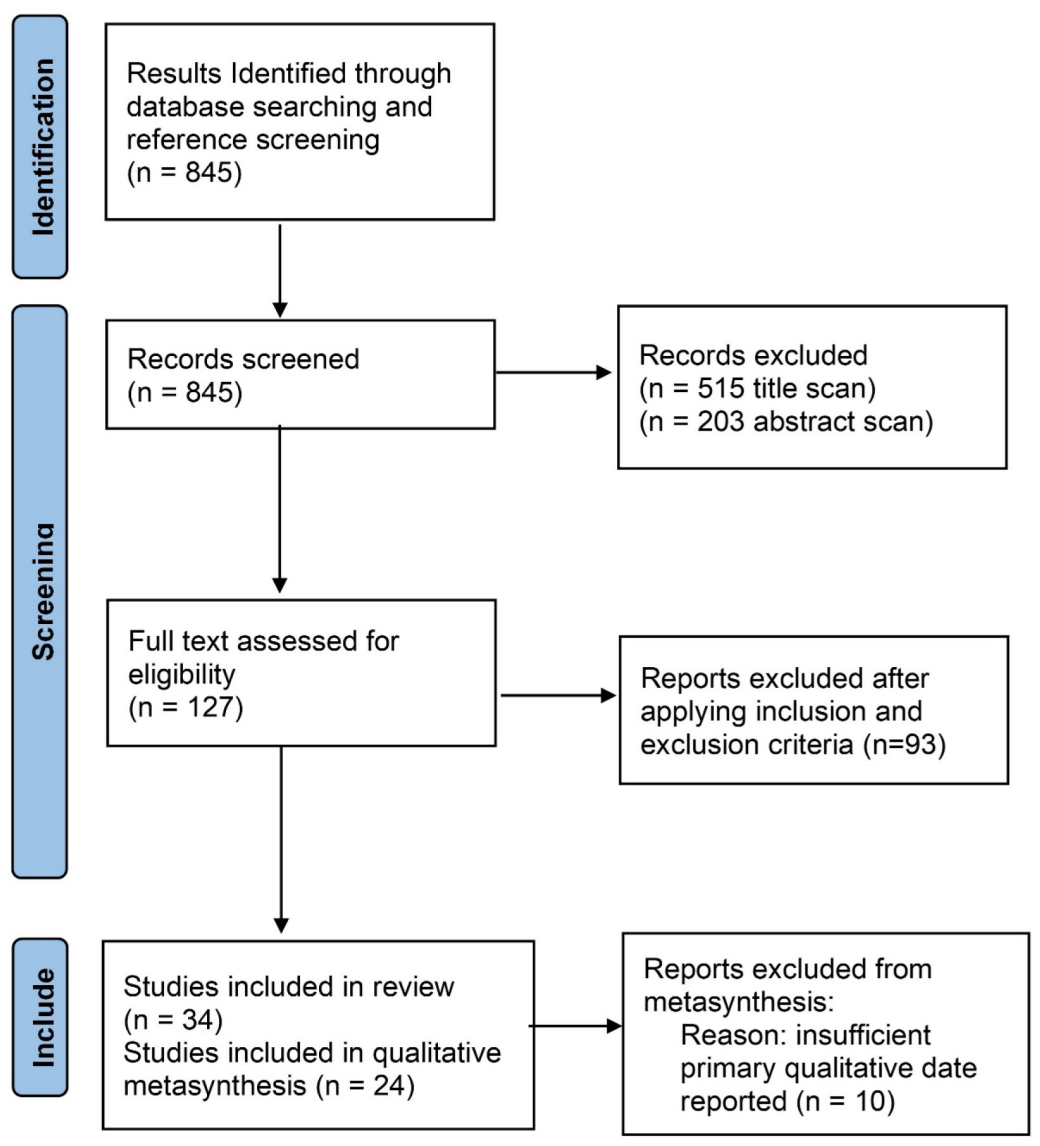
Prisma (2020) flow diagram of studies included.

This refinement produced *n* = 845 records for screening. Figure 1 shows the PRISMA flow diagram for screening to yield the final analytic sample of *n* = 24 full text articles included in the meta-synthesis.

### Quality assessment, data extraction, and synthesis

The Ritchie and Spencer framework was used to guide data synthesis (17). Quality assessment was conducted using the Critical Appraisal Skills Programme (CASP) for qualitative studies. Studies were appraised using the Critical Appraisal Skills Programme tool (CASP) for qualitative research (18). Each article was independently assessed by two co-authors who applied three levels of the CASP criteria: met, partially met, not met. Results were discussed to achieve consensus-based rating of all articles among raters. See Supplemental Table S1 in supplementary material for the 10-item quality assessment criteria used.

Results of database searches were entered into a bibliographic software program, Endnote™ (19), for automatic removal of duplicates. Data extraction was performed jointly on 3–5 sample articles to reach consistency in extraction information and the coding process. All articles were coded independently by two of three authors and results and themes were discussed as a group to ensure reliability and consensus among raters for both screening and extraction stages. Iterative code refinement and additions were made, discussed, and consensus reached by all authors to inform the resultant themes and sample quotes. All data extraction and management were performed using Microsoft Excel. See Supplemental Table 2 for final codebook used.

Thematic synthesis was performed using an iterative approach. Team members coded, reflected, and met to discuss code formation, categorization, and emergent themes. Line by line coding (whole manuscripts) was performed of two selected manuscripts to develop clarity on the coding scheme. Categories were established using a hierarchical tree structure with following data-driven themes identified. For the final step of synthesis and analytical theme identification team members discussed the first and second order constructs (20). Reviewers identified and reached consensus on the final results grounded in the goals of the research question: describing lived experiences, identification of unmet needs, as well as potential community-based policies, programs, and interventions to address those needs of family caregivers. See Supplemental Table 3 for details of coding structure and resultant themes and Supplemental Table 4 for data extraction elements of the analytic sample. Synthesis reporting follows ENTREQ guidelines (21).

### Community-based case study application

In order to situate this meta-synthesis in the context of a local community-based organization CBPR methods were implemented to add to the relevancy and application of these findings to the development of community-based policy and program recommendations. One-on-one interviews and focus groups among community members visiting the local Council on Aging in Medford, MA were conducted. Additionally, a review of program offerings was performed in order to categorize the availability of existing community-based resources. This content analysis provided an opportunity to compare findings of unmet need versus existing, yet underutilized, to discern reasoning for perceived or genuine gaps in caregiver supports.

## Results

A final analytic sample of 24 articles were used for the metasynthesis. Methodological quality of all included studies was deemed sufficient following appraisal using the CASP checklist for quality appraisal. The three themes (Structural Barriers, Emotional & Relational Impact, and Caregiver Capacity & Identity) collectively highlight the multifaceted impact of caregiving on caregivers' lives. Together, they reveal that caregiving is shaped by more than just day-to-day tasks; it is influenced by broader structural and systemic barriers that limit access to support, while also carrying deep emotional and relational consequences. At the same time, caregiving affects a person's identity, sense of capacity, and ability to function in daily life. These themes underscore that caregiving is a complex and holistic experience shaped by interconnected external pressures, emotional challenges, and personal transformations.

### Theme 1: structural and systemic barriers to caregiving support

Caregivers described substantial barriers embedded within health policy, service availability, and care delivery systems. Financial strain was persistent, with many expressing difficulties affording outside help even when it was essential. One caregiver asked, “*How much is enough to save?”(**22**)* reflecting uncertainty and anxiety over long-term care expenses. Rural caregivers reported significant access challenges due to geographic isolation and service scarcity and drive long travel distances for basic care support.

Navigational challenges were also prominent. Caregivers described a lack of coordinated information pathways following diagnosis, often receiving medical instructions without referral to social or community resources. One caregiver reflected*, “There's not an instruction manual on the journey… you just have to figure this out on your own”* *(**23**)*. Limited institutional trust further intensified these challenges, particularly among caregivers from culturally and linguistically diverse backgrounds.

In contrast to reported barriers, caregivers who were able to identify supportive programs highlighted the value of structured community services, including adult day programs and peer support groups in acting as facilitators of supporting the caregiver role. These resources offered essential emotional respite, meaningful social engagement for care recipients, and reduced burden at home.

### Theme 2: emotional and relational impact of caregiving

Caregivers reported profound emotional consequences, including grief, anxiety, anger, guilt, and a sense of entrapment. Many described an ongoing process of anticipatory grief as they watched loved ones lose their abilities and identity. Feelings of social isolation were common due to caregiving demands and diminished social contact.

“*...Health concerns and the physical requirements are only one part of the equation and not even the hardest part. The most difficult thing to deal with is the mental and emotional toll it takes [on] a person to watch a loved one slowly slipping into infirmity and pain and not being able to do anything about it. I was prepared for the physical demands when my mother came to live with us, but I had no clue about how emotionally draining it would turn out to be”* *(**24**)*.

Relational dynamics evolved significantly. Several caregivers described a reversal of family roles “*I'm the mother and he's the child*, *(**25**)* while others noted the loss of emotional intimacy or marital companionship. For some, caregiving fostered new forms of closeness, described as a “*gift of caregiving,”* *(**26**)* reflecting emotional resilience amid hardship.

### Theme 3: caregiver identity, capacity, and daily functioning

Caregiving responsibilities were described as all-encompassing and evolving over time, requiring caregivers to assume multiple roles: health manager, legal proxy, financial coordinator, medication monitor, personal care assistant, and advocate. Many perceived caregiving as a moral duty rooted in family values or faith traditions.

Caregivers worked to maintain routines and household stability despite rising complexity of needs. However, this often came at a personal cost: declines in physical health, neglect of preventive care, disrupted sleep, and increased psychological stress. Social participation decreased sharply, and caregivers struggled to sustain hobbies, exercise, or self-care. Even those with a positive outlook acknowledged the emotional and physical toll of caregiving.

### Integration of findings withing a community-based application

Findings from the program review illustrate that many domains of support are represented in existing program offerings (see Table 1). However, accessibility and utilization of these services among active family caregivers were limited. Opportunities to engage directly with active caregivers were limited; therefore, our findings draw primarily from interviews with former caregivers (n= 17 semi-structured interviews) and program administrators (2 in-depth interviews). Participants highlighted several significant concerns during the discussion. Many described a lack of flexibility in their daily lives, explaining that as caregivers they always felt they had to “*be at the ready”* which left little room for personal freedom or spontaneity. Others emphasized a shortage of resources, noting that there were “*not enough funds to travel or do other activities,”* and that even when resources were available, they were often “*too busy”* or simply did not have the time to participate in restorative activities. Several participants also spoke openly about their own physical and health limitations, including mobility challenges, a history of stroke, and other chronic conditions that made daily life more difficult. Beyond these practical challenges, many expressed a deep emotional need for greater grief support as they transitioned out of caregiving roles—often after the loss of the person they had been caring for. This phase of life, they shared, could be isolating and emotionally complex, underscoring the importance of supportive services and community understanding. Together, these findings illustrate that there are many resources available, yet barriers persist limiting caregiver ability to access services and supports related to unmet needs described in the meta-synthesis findings.

**Table 1:** Local council on aging community program supports available.

Program support area	Activity
1. Social and community engagement	Large (non–religious) celebrations
	Large (religious, cultural) celebrations
	External Social Gatherings (i.e., casino)
	Day/Half–Day Long Excursions
	Games (Bingo, Dominoes, etc.)
	Political Engagement Opportunities
2. Arts and creative expression	Art Opportunities (Choir, Theater)
	Creative Art Opportunities (i.e., knit)
3. Education and lifelong learning	Educational (non-health) Opportunities
	Health Education Opportunities
4. Health and wellness	Exercise Opportunities
	Screenings (ex. blood pressure screenings)
	Affinity Support Groups (ex. Language groups, Memory cafes)
5. Practical and basic needs support	Shopping Trips (Grocery Store, etc.)
	Financial support (ex. free Tax preparation)
	Food Pantry

## Discussion

These findings reinforce caregiving as a critical public health concern with significant implications for health equity, aging policy, and community wellbeing. Caregivers play a central but under-supported role in the US. long-term care system, often filling gaps in formal health and social services. Yet the results show that systemic shortcomings to the extent to which our healthcare system and community-based resources are meeting the needs of caregivers and their care partners. Specifically in our work with community-based senior centers, we observed notably low utilization of services by caregivers. Opportunities to engage directly with active caregivers were limited; therefore, our findings draw primarily from interviews with former caregivers and program administrators. These stakeholders consistently reported that the siloed structure of existing programs and the lack of respite offerings hinder caregivers' ability to access resources and support outside the home. This suggests an unmet need for program models that adopt a dyadic approach—one that simultaneously addresses the needs of both the caregiver and care recipient. Designing programs from a dyadic perspective may enhance participation by allowing caregivers and their care partners to engage in services together, thereby promoting shared benefit and improving overall accessibility and utilization.

Additionally, the structural and emotional barriers identified in this study have direct consequences for care quality and health outcomes. High caregiver strain is associated with worse self-rated health, increased hypertension, depression, and delayed medical care among caregivers (27, 28). When caregivers lack guidance, training, and respite—as demonstrated in our findings—the quality and safety of home-based care declines, increasing the likelihood of medication mismanagement, delayed treatment decisions, and preventable safety risks (29). These caregiving challenges also negatively affect care recipients: inadequate caregiver support is linked with accelerated decline in functional ability, behavioral symptoms, preventable hospitalizations, and early nursing home placement (30, 31). Furthermore, a robust body of research, grounded in a socio-healthcare perspective, emphasizes the importance of preventive strategies aimed at reducing emotional risks, such as depression, and mitigating physical decline. Quality-of-life indicators, including physical, mental, and psychosocial wellbeing, are positively influenced by structured interventions, particularly multicomponent or cognitively integrated exercise programs, which improve functioning within the patient–caregiver dyad and reduce caregiver burden (32). Moreover, accessible and well-structured programs that are tailored to the family context consistently demonstrate higher effectiveness and greater adherence (33).

These effects due to the “hidden” costs of informal caregiving carry significant health system consequences. Informal caregiving substitutes for the largest source of long-term care in the US., and its collapse due to burnout or lack of support shifts care to higher-cost institutional and emergency settings (28). Recent analyses show that caregiver burden contributes to increased Medicare expenditures through avoidable emergency visits and hospitalizations among dementia patients (30). The economic value of unpaid caregiving is significant, yet caregivers receive little formal training or systemic support (28). Sustaining caregiving capacity must therefore be treated as a public health priority to prevent downstream system strain and maintain care continuity for aging populations.

Overall, these meta-synthesis findings contribute to deeper understandings about the need to shift our approaches to a dyadic approach in policy, research, and practice to more holistically capture the dynamic nature of caregiver roles, responsibilities, and impact on overall quality of life for both the caregiver and care recipient. Furthermore, these findings highlight gaps in public health approaches to program development and implementation in that that limitation may not be the services available but rather the ability for caregivers to access these services (knowledge gaps, logistical challenges, accessibility issues, or competing responsibilities) that play a significant role in limiting caregiver utilization of community-based programs and services.

### Public health implications

Caregiving demands affect caregiver health outcomes, workforce participation, and community stability. Unmet caregiver needs lead to higher emergency care use, premature institutionalization, and caregiver burnout, each of which carries significant human and economic cost. Caregiving must therefore be integrated into public health programs and policy alongside complementary initiatives supporting chronic disease prevention or behavioral health.

#### Leveraging community-based supports

Study findings highlight a disconnect between available services and caregivers' ability to access and trust them. Community-based programs—local senior centers, Councils on Aging (CoA), Area Agencies on Aging (AAA), adult day health programs, faith-based networks, and intergenerational programs—are uniquely positioned to close this gap. The study provides evidence for the following recommendations to expand community-based caregiver supports:

*Strengthening Navigation Support Pathways*

Caregivers urgently need individualized, community specific information. Embedding care navigators within councils on aging or senior centers, or via social prescribing pathways in the clinical setting, could reduce system complexity by offering personalized care plans, resource referrals, and benefits counseling (34). Ensuring flexibility in program delivery models may foster community-centered, sustainable models that extend beyond current models that focus primarily on health services navigation but also to include social and community-based needs and supports that were highly represented in the findings.

*Expand Adult Day and Respite Services*

Adult day services can reduce caregiver burden and are associated with increased quality of life outcomes but utilization of and access to these services are limited by high cost, geography (more often limited to urban areas), and availability of hours and capacity (35). Public health investment must expand Medicaid-covered respite care options, accessible transportation support in rural and urban areas, and culturally tailored adult day programs.

*Normalize Peer Support as Preventive Care*

Support groups provided emotional coping, disease education, and reduced isolation (36). Integrating caregiver support programs—whether delivered in-person, remotely, or through hybrid modalities—into established community settings such as public libraries, local Councils on Aging, or intergenerational partnerships with schools and universities represents a promising strategy for extending the reach of evidence-based caregiver interventions within existing community infrastructures. Leveraging remote-based peer support options, centered in one's home offers opportunities to circumvent logistical obstacles many caregivers report experiencing. Lastly, by embedding these services within routine community programming may help to normalize caregiving roles, thereby reducing stigma and mitigating a key interpersonal barrier that prevents some caregivers from seeking needed support.

*Prioritize Culturally Responsive Aging Support*

Caregivers from diverse communities described cultural mismatch and mistrust in utilizing formal caregiver support services. Community-based organizations should partner with local senior centers, faith institutions, and community health workers trained to deliver linguistically and culturally responsive care navigation. Research and support for innovative culturally-centered programs is sparse (37). Continued work that leverages community-based participatory research (CBPR) approaches for both research and program development offers a critical opportunity to address existing gaps in the evidence base, policy, and practice, thereby supporting the increasing diversity of caregivers across the lifespan.

*Integrate Caregiver Wellness into Program Design*

Caregivers reported that they repeatedly neglected their own health. Senior centers and aging councils can become wellness hubs, offering caregiver health screenings, stress management services, respite-linked fitness programs, at-home suggestions for movement, and mental health referrals. Investing in community-based caregiver programs and preventive interventions can mitigate these downstream costs, preserve family stability, and reduce strain on the healthcare system. Advocating for comprehensive policy reforms and expanded insurance coverage to integrate caregiver support into routine healthcare encounters is essential for early identification of health declines and prevention of long-term complications. Implementing a dyadic model of care—one that addresses the needs of both caregivers and care recipients—offers potential to enhance sustainability of caregiving roles as families age together.

### Limitations

While this study includes a robust set of literature, a few limitations should be noted related to the metasynthesis finding. This study focuses on older caregivers living in the United States. As such the findings may vary when applying these recommendations to other countries or cultures. Furthermore, when synthesizing and extracting data from primary qualitative studies, there is always a challenge in balancing the preservation of the primary data richness with the goal of abstracting and synthesizing across the larger body of work. As mentioned previously, our ability to interview active caregivers within the local senior center was limited; therefore, our findings draw primarily from interviews with former caregivers and program administrators. Furthermore, our applied context was limited to one community-based senior center in Massachusetts. While there are many similarities across senior centers, the generalizability to other regions or centers serving different populations with differing resources may be limited.

## Conclusion

To meet the needs of our aging population, caregiving must continue to be positioned as a key public health priority. The caregiving experience is shaped by a complex array of structural limitations that extend beyond individual-level factors based on caregiver and care partner characteristics alone. Public health systems must recognize caregivers as a vital extension of the care continuum and invest in robust, community-based infrastructure that sustains their capacity. Senior centers, Councils on Aging, and local community partners can serve as community-based resource hubs, transforming caregiving from a private struggle into a supported social role that preserves dignity, health, and connection for families aging together.
