# Supplementary material for: Caregiver support in aging societies: a qualitative metasynthesis informing public health policy
Source: Front Public Health. 2026 Jun 11;14:1821540. doi: 10.3389/fpubh.2026.1821540 (PMC13293888; doi:10.3389/fpubh.2026.1821540)
Supplement: Supplementary file 1 [file Table_1.docx]

**Supplemental Table S1: Critical Appraisal Skills Programme criteria**

| **CASP item** | **Criteria used** |
| --- | --- |
| 1. Was there a clear statement of the aims of the research? | Aim can be determined from anywhere in the paper (e.g. title, abstract, introduction, methods) |
| 2. Is a qualitative methodology appropriate? | Qualitative research is the appropriate way to address the aim or answer the research question (e.g. the research concerns experiences/views of participants, processes involved, or the nature of interactions) |
| 3. Was the research design appropriate to address the aims of the research? | Data collection, data analysis and methodological approach are appropriate to address the aim or answer the research question; the review team classified methodological approach based on whether the approach had consistently been used throughout (e.g. grounded theory required analytical methods such as constant comparison as well as the development of a core category or theory) |
| 4. Was the recruitment strategy appropriate to the aims of the research? | Authors clearly report selection of participants and this is appropriate to address the aim or answer the research question. |
| 5. Was the data collected in a way that addressed the research issue? | Type of data collection method is clear (e.g. interview, focus group, participant observation, online postings) and has potential to address the aim or research question. |
| 6. Has the relationship between researcher and participants been adequately considered? | Authors demonstrate self-reflexivity in data collection and data analysis processes |
| 7. Have ethical issues been taken into consideration? | Approval from an ethics committee is reported |
| 8. Was the data analysis sufficiently rigorous? | Authors clearly describe data analysis approach and employ strategies to ensure rigour (e.g. peer debriefing, member checking, negative cases). |
| 9. Is there a clear statement of findings? | Findings are clearly stated (e.g. in the abstract or findings) |
| 10. Is the research valuable? | Study appears valuable in own right (i.e. something has been ‘learnt’), rather than judged in relation to contribution made to the current review. |

**Note:** Each criteria were scored [Met, Partially Met, Not me]t. Each study was independently appraised by 2:3 authors. Finral appraisals were discussed by all members of the study team to obtain consensus regarding final appraisal rating for the overall manuscript included in the study.

Source: *CASP Qualitative Checklist*. URL: [www.casp-uk.net/#!casp-tools-checklists/c18f8](http://www.casp-uk.net/#!casp-tools-checklists/c18f8)
