# Supplementary material for: Caregiver support in aging societies: a qualitative metasynthesis informing public health policy
Source: Front Public Health. 2026 Jun 11;14:1821540. doi: 10.3389/fpubh.2026.1821540 (PMC13293888; doi:10.3389/fpubh.2026.1821540)
Supplement: Supplementary file 2 [file Table_2.docx]

**Supplemental Table 2: Codebook**

| **Code Name** | **Code Definition/Description** |
| --- | --- |
| Uncertainty | Expressing unknown about future needs, care, responsibility |
| Lack of Knowledge | Not knowing what to do, unable to find resources/support, looking for answers from others |
| Skepticism/Downplaying Issues | Thinking everything is ok, denial of needs, attributing it to “normal aging” |
| Level of Responsibility | The degree to which the caregiver feels responsible for the care recipient—may vary from low (little responsibility to higher but the content refers to feeling responsible |
| Role Transitions | References to changing the relationship or roles over time |
| No changes in relationship/role | Specific language capturing feeling that nothing has changed |
| Need for Monitoring | Content focuses on need for monitoring safety or other oversight tasks |
| Taking on a Managerial Role | Moving from a partner to more of a manager—of daily activities, community outings, medications, etc. |
| Taking it all on | Referencing the all-encompassing aspect of caregiving, “doing it all” |
| Use of outside support | References to the use (or lack of) outside supports. This may be formal care services, programs, or informal community supports |
| Extensive Care | Capture the scope of care and severity of needs |
| Disapproval of outside support | Focuses on aspects of mistrust or rationale for not using outside supports |
| Duty to provide care | Providing care is a fundamental duty or responsibility as a family member |
| Family dynamics of decisions | Captures the complexity of decision making may include the care recipient or other family members |
| Trust drives care role | Content that specifically references aspects of trust and how that is important for the caregiving role |
| Reflections include pre-caregiver timeframe | Degree to which caregivers are comparing life before the caregiving role began |
| Changes induce anxiety/fear | Specific reflections and feelings about how the caregiver feels anxious about changes/future |
| Feeling frustrated, unheard | Content references caregiver frustration with not feeling heard, needs unmet when expressed to others, lack of support from providers (ex. Clinicians not listening) |
| Putting life on hold | References to total shift of lifestyle, this may also include reference to the care recipient as part of this as well...stopping usual activities to now be a caregiver or attend to care recipient needs |
| Feelings of grief/loss/sadness | Content captures feelings of sadness, grief, loss from the caregiver as part of this role/situation |
| Usefulness of support groups | Specific reference to several types of support groups (can also include not using them) |
| Coping strategies | Content that describes ways the caregiver copes or tries to maintain their own health or attend to their own interest/needs |
| Sense of community and purpose | References a positive aspect of caregiving providing a sense of purpose and opportunity to share experiences with others |
| Social isolation | Content reveals aspect of the caregiver feeling isolated |
| Desiring an end | Specific references to desiring an end to the role (this may be on behalf of the care recipient) but highlights that the caregiver feels down about the future trajectory |
| Changes in community participation | Reference to changes in how the caregiver (or caregiver/care recipient dyad) participate in community activities, may include perceptions of community members limiting welcomeness or not accommodating to community participation |
| Compromising own health | Forgoes own health, self-care, sleep, etc. to attend to others needs |
| Caring requires patience and flexibility | Mentioning need to be flexible, patient, good listener given the complexity of needs of the care recipient |
| What is health | Reflects caregiver perspective of what health/well-being is for themselves (ex. Eating well, exercising, maintaining social well-being) |
| Health decline since caregiving | Noting how health has declined (physical, mental, or overall well-being) since assuming the role of caregiver |
| Feeling trapped | Comments reflecting lack of choice, no other options, feeling trapped as a primary caregiver |
| Factors that would help | Services or strategies that would help overall caregivers’ lives; potentially reduce caregiver burden |
| Relationship dynamics | Importance or expressing specific nature of the relationship and how that may affect the caregiving role (ex. Daughter vs. Spouse) |
| Multiple roles beyond caregiver | Data showing other responsibilities (caring for other children, work, etc.) that are beyond the caregiver role providing care for the person living with AD/ADRD |
| Ability and desire to care at home | Notes increasing difficulty in caregiving role, often does not want to use institutional options but not sure of ability to continue as primary caregiver |
| Research served as interaction | Several studies participants mention the process of the research interview being therapeutic and they enjoy the social aspect of the interview—this could indicate issues with social isolation |
| Language/Cultural Barriers and Drivers | Reflecting difficulty in accessing or receiving high-quality care due to obstacles with language use or comfortability in the way care is delivered (i.e., cultural belief that care should be delivered in the home vs institutional environment) |
| Affordability/Financial Supports | Comments reflecting challenges in covering the cost of care or caregiving resources, as well as issues in locating potential financial support available to them. |
| Actions driven by guilt/regret | Mentioning that caregiving for an individual was driven by a desire to avoid guilt or regret after that person had passed. |
| Acquiring knowledge of care/Not knowing of outside support/Self-doubt of abilities | Not feeling properly resourced or trained in delivering care, and expressing uncertainty as to where that information can be obtained credibly; Not feeling confident in providing care sufficiently |
| Legal Barriers to Caregiving | Not possessing legal decision-making power for the individual being cared for, leading to unnecessary delays |
| Loss of Intimacy | Acknowledging a key possible challenge of caregiving, particularly for a spousal partner, is losing the ability to be intimate. |
| Seeking to maintain status quo/Routine-building as a critical component of caregiving | Despite changing physical or psychological conditions of the condition, a desire expressed by the caregiver to maintain prior household roles and responsibilities |
| Rural-specific obstacles | Highlighting specific barriers in accessing care for rural-based populations |
| Sentiments of denial | Not acknowledging the reality of the changed/changing condition of the person being cared for |
| Positive elements of caregiving | Highlighting positive characteristics of the caregiving experience like being able to spend considerable time with loved one. |
| Mistrust in health system | Expressing a lack of trust for the health system to deliver their loved one high-quality care/negative experiences in health system |
