# Supplementary material for: Caregiver support in aging societies: a qualitative metasynthesis informing public health policy
Source: Front Public Health. 2026 Jun 11;14:1821540. doi: 10.3389/fpubh.2026.1821540 (PMC13293888; doi:10.3389/fpubh.2026.1821540)
Supplement: Supplementary file 3 [file Table_3.docx]

**Supplemental Table 3: Coding Structure and Theme Results with Exemplar Quotes**

| Theme | Subtheme | Supporting Codes | Exemplar Quotes |
| --- | --- | --- | --- |
| Theme 1: Structural and Systemic Barriers to Caregiving Support Health policy, service infrastructure, and systemic limitations that shape caregivers' ability to access and trust support. | Access and Affordability | Affordability/Financial Supports - Rural-specific obstacles - Legal Barriers to Caregiving - Use of outside support - Disapproval of outside support | “Would we be able to afford to have anything (now)? I only thought of future care as money. That’s my primary concern. That’s –And, if we would be able to afford, would it be just for one person? Or is there enough for both of us? I mean, we’ve saved our money, but how much is enough to save?” (Mayo et al.)  “Where do we find help in rural ND? I have put almost 250,000 miles on my car in 6 years driving to medical appointment . . . There needs to be more services available in rural ND. We are a rural state, but most services are located in the 4 bigger cities. We are over 2 hours away from any of them” (Strommen et al.).  “Adult day care really saved our family life. . . . I completely credit them with giving her a sense of purpose in life. She says she works there; she’s going to the shop. In the evening if you ask her what she did, she said, “Well, I did piecework and patterns, and you know all that stuff.”. . . She just loves it and I think their formula has just worked really, really well” (Czekanski).  “There’s not an instruction manual on the journey, but it would have been good to have some guidance and someone to help facilitate this journey. Like after the neurologist diagnosis if someone said, here are local resources in your area, here’s where you go for help with power of attorney, here’s how to get help with in-home care. Anything like that—but you just have to figure this out on your own. Instead, we were just told the medications to take. There was no follow up. I didn’t feel like I had anyone to turn to. There needs to be a more social services end of it, someone who would have a better idea of what the patient and I would go through” (Duplantier & Williamson).  “I can’t leave him alone. I could only go when that aide was here. It would have to be somebody I felt I could trust that wouldn’t rob me blind while I’m gone. You just can’t, well, I need this. I’ll just zip up and run to the store. No, you’ve got to wait” (Richards et al.).  “It’s not the same having him here at home versus having him at a center or…or a nursing home…they don’t offer the same kindness that is offered here at home…” (Jaldin et al.). |
|  | Institutional Trust and Navigation Challenges | - Mistrust in health system - Lack of Knowledge - Acquiring knowledge of care / Not knowing of outside supports / Self-doubt of abilities - Research served as interaction - Language/Cultural Barriers and Drivers | “I had a lot of difficulties because I had no experience with taking care of someone with Alzheimer’s before him. Sometimes he remembers, and sometimes he does not” (Nguyen et al.).  “I kept calling, "How are you treating her if no one knows her background?" No one cared. It was a nightmare. I kept calling different departments, trying to get somebody to help…That’s how COVID affected us…I was not allowed in…There were no exceptions…There was no one to advocate for my mom” (Yan et al.).  “Caregivers feel overwhelmed when they are not confident in providing care and treatment. There is sometimes a lack of education about the condition of the care recipient, and how to address their needs. They could use more education and reassurance from the medical providers to let them know if they are doing a good job. They need the medical providers to ask for their opinion more often—sometimes people are discharged even though the caregiver is not comfortable, because the caregiver isn’t given the opportunity to talk about concerns, or doesn’t feel comfortable doing so” (Strommen et al.).  “There’s not an instruction manual on the journey, but it would have been good to have some guidance and someone to help facilitate this journey. Like after the neurologist diagnosis if someone said, here are local resources in your area, here’s where you go for help with power of attorney, here’s how to get help with in-home care. Anything like that—but you just have to figure this out on your own. Instead, we were just told the medications to take. There was no follow up. I didn’t feel like I had anyone to turn to. There needs to be a more social services end of it, someone who would have a better idea of what the patient and I would go through” (Kim et al.).  “You need support that is practical. Someone that goes to your house and guides the family with useful information, in a practical and real way. Sometimes you need in-person support, to be able to talk to someone who can tell you how things really are with Alzheimer’s. About the services, and what they are” (Martinez et al.).  “There’s no facility, or at least I don’t know yet, that has large Korean-speaking programs … that also all the staff is well-trained. I didn’t find a place like that…. [I] actually went to visit two facilities. It’s all Korean daycare centers run by Korean staff. They speak Korean language and provide Korean food, which is a lot of comfort. But both of them are really crowded…. [Without Medicaid] you have to pay privately…. I wasn’t sure about the quality of their real program. ” (Kim et al.). |
|  | Support Structures and Community Integration | - Usefulness of support groups - Sense of community and purpose - Factors that would help | “I would encourage anyone to immediately find a group close to where they live and go to it. . . . Start building a good support system around you” (Czekanski).  “So, I think we need a good affordable type of group or organization or funds for middle-income people who are still struggling and need that additional support monetarily so that their loved ones can also get the assistance they need” (Ramos et al.).  “I would encourage anyone to immediately find a group close to where they live and go to it. . . . Start building a good support system around you” (Duplantier & Williamson).  “​​I think cruising is ideal for people with memory problems,” (Mrs. Williams, spouse, high couple hood) due to the safe and enclosed nature of vacationing on a cruise ship. This exemplifies the effort spouses put in to meet their loved one with AD “where they are” and to modify vacations to better suit their spouse's abilities as opposed to simply ceasing the activity” (Gallagher & Beard).  “ …And I take him to [a day program]… He goes there on Tuesdays for half a day. And I have a volunteer that comes once a week…I can’t complain about support group. I have plenty of support” (Hazzan et al.).  “I get very frustrated…And sometimes I get very angry at her. I’d want her not to be that way. I’d want her to listen to reason, and what I learned in my group is the little motto was BTD which is ‘blame the disease’ (Meyer et al.). |
| Theme 2: Emotional and Relational Impact of Caregiving on Older Adults Psychological and relational dimensions of caregiving, including grief, identity shifts, and complex interpersonal dynamics. | Emotional and Psychological Strain | - Uncertainty - Feeling trapped - Feeling frustrated, unheard - Putting life on hold - Changes induce anxiety/fear - Desiring an end - Actions driven by guilt/regret - Sentiments of denial - Feelings of grief/loss/sadness | “I just feel so badly you know for the grandfather he could of been and what he is going to miss out on. You know I mean he’ll certainly be here and love a grandchild, but it won’t be the same… I just think you know the grand fatherhood he was supposed to have has been stolen from him” (Meyer et al.)  “I anticipate that in the next few months he will pass. In some ways I’ve been coping with it for years… so I anticipate that when that happens there will be a combination of PTSD and relief” (Gallagher & Rickenbach).  “Accepting the disease is the most difficult. When the deterioration begins, they become a different person, so then it’s hard for you to understand that right where this monster is, used to be a person … sometimes you tell yourself, “My God, why do I have to do all of this?” (Martinez et al.).  “It makes me feel like everyday life is uncomfortably constantly changing and I don’t have any way to predict what’s coming. Everything is so uncertain. It is the role of the caregiver, unpredictable and then on top of that, the COVID is also unpredictable” (Richards et al.).  “So, it’s a frustration of what I perceived the normal living, where we would do things together. We traveled together before all this. I hate to use the word frustrating, but it’s just very frustrating to try to be the cheerleader all day long, because some days I have bad days too. So, it’s just a whole—There’s that word frustrating again” (Mayo et al.).  “About two years ago … I did occasionally have those thoughts…. I imagined hanging myself, but then again didn’t know what to do about my husband. I even imagined going on a cruise and jumping off together…. The idea of disappearing off the face of the Earth didn’t seem so bad” (Kim et al.).  “I feel most of my friends and family are able to come and go as they please and do what they need to do and take care of themselves, and I don’t have that option. I’m kind of trapped” (Hazzan et al.).  “...Health concerns and the physical requirements are really only one part of the equation and not even the hardest part. The most difficult thing to deal with is the mental and emotional toll it takes [on] a person to watch a loved one slowly slipping into infirmity and pain and not being able to do anything about it. I was prepared for the physical demands when my mother came to live with us, but I had no clue about how emotionally draining it would turn out to be” (Wang).  “My husband is now spending a lot of time in bed. He doesn’t want to get up and get dressed. So, unless I can get someone to come over and be here, I can’t go anyplace or do anything. I feel like a prisoner. It’s not a 12 x 12 room but it is solitary confinement” (Duplantier & Williamson).  “I just felt that I was too young, there were so many changes. I didn’t really want to. . . . It’s almost like you have to put your life on hold. I had no idea how long that it’s going to be. I remember . . . I just told her, “Let’s just go into the garage and turn the car on and just end it” . . . rather than go through it. I guess I was trying to feed her an idea. Isn’t that awful?” (Czekanski). |
|  | Interpersonal Dynamics and Role Evolution | - Role Transitions - No changes in relationship/role - Loss of Intimacy - Relationship dynamics - Family dynamics of decisions - Trust drives care role - Reflections include pre-caregiver timeframe | “I’m the mother and he’s the child, and that’s just the way I view it. I don’t have a husband; I don’t have a mate.” She also said, “Sometimes I think I’m the maid with the cleaning him up, with the making the meals, doing his clothes, doing his laundry” (Czekanski).  “So, it was a different relationship and we were very, very close. We had a very good physical relationship, a good emotional and spiritual relationship so it was a real marriage and I think when you take care of somebody too, you know, when you had to wipe their bottoms and help them bathe and do all the things you do, there’s an intimacy there that doesn’t, probably doesn’t even exist if you haven’t been the caregiver. And that’s the gift of caregiving, I think. You create a new kind of intimacy” (Hovland et al.)  “My mom got sick a year after my father died. I was going through a grieving process for that and then I went into a grieving process for her. It has been a lot of grief. The roles change—they become the child and you become the parent—it’s very confronting” (Duplantier & Williamson).  “We've had a very good relationship. Um, and uh, so that's gonna change. I mean it's gonna change in that I become more of a nag. Constant reminders change the nature of the relationship” (Gallagher & Beard).  “I describe it like what happened to the husband I had. It's totally a different person, unable to do things for himself, unable to think on his own, and unable to go out and enjoy himself like he used to, and it's just hard because I've known him for so many years and all of sudden he's a different person, another person, another personality I have to get accustomed to. It's very difficult, very, very difficult at times” (Guerrero & Mendez-Luck). |
| Theme 3: Caregiver Identity, Capacity, and Daily Functioning Day-to-day experiences of caregiving, caregiver capacity, and how caregiving responsibilities evolve and intersect with caregivers' own well-being and agency. (Transitional in nature) | Caregiver Role Complexity and Load | - Taking on a Managerial Role - Taking it all on - Duty to provide care - Extensive Care - Multiple roles beyond caregiver - Level of Responsibility | “.... So, I’ve taken on everything. Everything falls in my lap. I am the one that has to see the bills are paid and whatever comes up. It’s all on me’ (Potter et al.).  “Even when my mom lived in Nashville in her own condo, [or stayed with my brother on the East Coast], I felt responsible for everything” (Duplantier & Williamson).  “There is a hierarchy, everybody has a position in the family and that’s what they taught us and that’s what we do.... I find more as a Black people in general, that’s what we do, we take care of our people.” In addition, another family member said, “To be honest it’s like something I got to do; I mean God put us here, our parents took care of us, sometime we got to take care of them” (Epps et al.).  “As long as we can, we're gonna take care of each other. And we're gonna stay home” (Duplantier & Williamson).  “While I’m still alive and have the strength to do it, I’m going to take care of her.’ Caregiver 4 (Latina female, 66 years old, caring for parents) said, ‘…we take care of our own and that’s the way it goes” (Jaldin et al.).  “My husband is no longer driving. And I’m the number one transporter. He’s no longer taking his own medicines due to double dosing himself. And so, I became a pharmacist. He’s no longer able to make any of his own meals. He’s not able to operate a microwave. And so, I’m chief cook and bottle washer.” (Hazzan et al.).  “Well, I have to provide--I have to assist him showering, brushing his teeth, dressing. So, I drive him everywhere. I keep track of all his appointments, and I just totally take care of him. I’m just gonna do what I have to do and find out and research” (Mayo et al.)  “For me, my mother is not an obligation, she’s, my responsibility. If one has to do things out of obligation, they won’t be happy doing it. My family believed that because I was a nurse, I could take care of her. But that did not mean that I had the obligation to … I had the love and dedication” (Martinez et al.). |
|  | Maintaining Control and Routine | - Seeking to maintain status quo / Routine-building as a critical component of caregiving - Caring requires patience and flexibility - What is health | “Caring requires patience. We cannot apply logic and explain things to them. If they say 1, then we agree that’s 1; then they will be happy. We don’t go against their will. We know their perspectives are absurd, and it can be frustrating, but we have to calm down as it can affect us and make us exhausted. They can misplace something and blame and accuse us of hiding it from them. In the past, I would argue with him (husband). Now, from experience and through time, I just go with the flow” (Nguyen et al.).  “Well-being looks like happiness. Someone is always checking in on themselves and making sure they are in a good place. They make sure they are doing things that brings them joy, things for yourself” (Duplantier & Williamson).  “I think another thing was just having a routine for my grandma. Because when things happened that weren’t in her normal routine that seemed to either make her way more anxious or way more aggressive, depending on where in her illness she was. So, the more that we could do that, just stayed in a routine pattern the smoother things seemed to go” (Polenick et al.). |
|  | Health and Social Participation | - Health decline since caregiving - Compromising own health - Social isolation - Changes in community participation - Ability and desire to care at home - Positive elements of caregiving - Skepticism / Downplaying Issues | “I’m fundamentally a glass-half-full kind of person so we have had a long life of a good marriage, we have great kids, we have financial security, we have good health care. You know the fact that he has this illness sucks, it sucks for him more than it does for me, and I try to be grateful for every day” (Gallagher & Rickenbach).  “And then…I wasn’t doing my same exercising or taking care of my eating…it was kind of because I was putting more into them, I think, worried about them more. And then, I ended up…about 3 weeks ago…I had emergency surgery…” (Richards et al.).  “…when we used to go a lot of places with people, we don’t get invited anymore. And I blame it on that because he doesn’t talk much. …and if he says something then I see their eyes roll. I think they’re uncomfortable. Sometimes I’m uncomfortable when I see the eyes rolling. But I guess that’s the biggest thing, being excluded from everything” (Mayo et al.)  “I noticed myself being more depressed…. After some time, I thought I should do something about it and told the doctor about my depression, and he suggested I try taking some medication. I thought being around my husband all the time might negatively impact my emotional health…The morning after I took the medication, I had the weirdest experience looking at myself in the mirror. It was definitely my face, but it wasn’t my usual face. Even my daughter told me that day that my facial expression and affect was not of my usual self” (Kim et al.).  “I kind of forgot myself though, but that does happen. I wasn’t taking care of my diabetes. I wasn’t taking my medication on time. I wasn’t eating right. I wasn’t sleeping, and that’s what brought [on] one of the heart attacks...too much stress. I wasn’t getting out in order to be here to care for him” (Guerrero & Mendez-Luck).  “...And it’s hard we’ve lost most of our friends.” We have one set of friends that still gets together with us… They’ve kind of given up on us…we tried to go out with two other friends, and they didn’t think about the fact that there were steps to get up to the place where we are going to eat and it was hard for him…” (Hazzan et al.)  “Before my husband got sick, I used to jog all the time. I love to jog. I haven’t been able to do that in over a year and my chubby body is a proof of it… it’s a question of time…you can’t leave him alone… ” (Hazzan et al.).  “…I have deferred personal health care because of concerns for continuity of care for my wife. My current concerns are exercise, weight management, benign prostatic hyperplasia (BPH) evaluation, and probable surgical intervention” (Wang et al.).  “The grief that I was experiencing after my mom moved was from the letdown of what I had been going through for the last ten years, combined with COVID. I was drinking like, I mean, not enough to say you’re an alcoholic or something, but for me, way more than I ever had. And that led me to just eat, you know, more unhealthy food. You know, you drink and then you eat badly, then you just feel gross and then you eat more gross food, and the whole thing just gets out of whack. Then your sleep starts to be disrupted” (Duplantier & Williamson).  “With them it’s been really hard. She’s a social butterfly. And not seeing anybody except us, you know, and then the caregiver that comes in, it’s hard on her” (Britt et al.). |
