# Supplementary material for: Caregiver support in aging societies: a qualitative metasynthesis informing public health policy
Source: Front Public Health. 2026 Jun 11;14:1821540. doi: 10.3389/fpubh.2026.1821540 (PMC13293888; doi:10.3389/fpubh.2026.1821540)
Supplement: Supplementary file 4 [file Table_4.docx]

**Supplemental Table 4: Final Analytic Sample and Preliminary Data Extraction Information Form**

| **Title** | **Author** | **Year** | **Sample** | **Setting** | **Method** | **Phenomena of Interest/Goal** | **Data Analysis** |
| --- | --- | --- | --- | --- | --- | --- | --- |
| Overcoming a Bad Day: A Qualitative Look into the Dementia Caregiving Experiences of Mexican-Origin Women in East Los Angeles | Guerrero LR, Mendez-Luck CA. | 2019 | N = 9 | Los Angeles, CA | In-depth, semi-structured interviews | inter-personal challenges faced by Mexican-origin female caregivers and their coping mechanisms within socioeconomic contexts | systematic and iterative data analysis through a modified grounded theory approach; iterative coding and data reduction |
| Family caregiver quality of life and the care provided to older people living with dementia: qualitative analyses of caregiver interviews | Hazzan AA, Dauenhauer J, Follansbee P, Hazzan JO, Allen K, Omobepade I. | 2022 | N = 23 | Rochester, NY; Chicago, IL | pre-interview | explore caregivers perceived quality of life in relation to the care provided to | qualitative content analysis |
| The influence of Latino cultural values on the perceived caregiver role of family members with Alzheimer's disease and related dementias | Jaldin MA, Balbim GM, Colin SJ, Marques IG, Mejia J, Magallanes M, Rocha JS, Marquez DX. | 2023 | N = 16 | Chicago, IL | individual, in-depth, semi-structured interviews | how Latino cultural values shape perceived caregiving experiences of middle-aged and older Latinos | descriptive statistics for demographics, codebook development |
| The impact of dementia caregiving on self-care management of caregivers and facilitators: a qualitative study | Wang XR, Liu SX, Robinson KM, Shawler C, Zhou L. | 2019 | N = 45 | South-Eastern USA | Semi structured, individual in person interview | investigate the influence of caregiving on the self-care management of dementia and AD caregivers based on the caregivers’ experience | thematic analysis (morse and field method) |
| Buffer or Blade: Perceived relationship closeness in couples navigating Alzheimer's | Gallagher E, Beard RL. | 2020 | N = 11 | Greater Boston Area | unstructured, in-depth interviews | understand how married heterosexual couples experience Alzheimer's | grounded theory, coding |
| Barriers and Facilitators of Health and Well-Being in Informal Caregivers of Dementia Patients: A Qualitative Study | Duplantier SC, Williamson FA. | 2023 | N = 8 | United States | semi-structured telephone interviews | identify barriers and facilitators to health and well-being for informal caregivers of family members with Alzheimer’s | reflexive thematic analysis |
| "I was Confused About How to Take Care of Mom Because this Disease is Different Everyday": Vietnamese American Caregivers' Understanding of Alzheimer's Disease | Nguyen H, Zaragoza M, Wussler N, Lee JA. | 2020 | N = 20 | Southern California | face-to-face semi-structured interviews | how Vietnamese American caregivers understand AD and provide care to family members | independent and co-coding |
| Religion, Spirituality, and Coping During the Pandemic: Perspectives of Dementia Caregivers | Britt KC, Richards KC, Radhakrishnan K, Vanags-Louredo A, Park E, Gooneratne NS, Fry L. | 2023 | N = 11 | Central Texas | Semi-structured, open-ended telephone interview | impact of social distancing on religious/spiritual practices and coping in dementia caregiver dyads from the perspective of caregivers | Content analysis using the vulnerability-stress-model’s framework |
| CE: Original Research: The Experience of Transitioning to a Caregiving Role for a Family Member with Alzheimer's Disease or Related Dementia | Czekanski K. | 2017 | N = 10 | Philadelphia, PA | two in-person interviews, guided by participants | explore lived experiences of people transitioning to caregiver role | phenomenological methods, peer debriefing |
| Decisions, Decisions: African American Families’ Responses to Mild Cognitive Impairment | Potter EC, Roberto KA, Brossoie N, Blieszner R. | 2017 | N = 27 | Midwestern and Southeastern US | Individual, in-person semi structured interviews | how diagnosis acceptance and family decision-making are at the heart of how AA families respond to mild cognitive impairment (MCI) | Iterative coding, thematic content analysis (but not explicitly stated) |
| Perceptions of couple hood among community-dwelling spousal caregivers | Gallagher E, Rickenbach EH. | 2020 | N = 13 | United States | Semi-structured interviews | better understand the experience of and changes in couple hood among spousal caregivers | Content analysis |
| How do family caregivers of older adults cope with relationship strain? | Meyer K, Rath L, Avent E, Benton D, Nash P, Wilber K. | 2023 | N = 75 | Los Angeles, CA | nine focus groups and interviews | studies of families' experiences with caregiving to older adults most often focus on overall burden and stress. | Thematic analysis |
| The Experience of Alzheimer's Disease Family Caregivers in a Latino Community: Expectations and Incongruences in Support Services | Martinez IL, Acosta Gonzalez E, Quintero C, Vania MJ. | 2022 | N = 24 | Miami, FL | Semi-structured interviews at home for caregivers with observational data collected | highlight the experience of care for Latino family caregivers to persons with Alzheimer's disease and related dementias (ADRD) | Grounded theory analysis; using comparative analysis strategy |
| "Please Don't Forget Us": A Descriptive Qualitative Study of Caregivers of Older Adults with Alzheimer's Disease and Related Dementias During the COVID-19 Pandemic | Richards KC, Radhakrishnan K, Britt KC, Vanags-Louredo A, Park E, Gooneratne NS, Fry L. | 2022 | N = 11 | South-Central US | Semi-structured, in-person interview; Follow up questions | impact of the COVID-19 pandemic on the overall well-being of older adults with ADRD living in nursing and private homes and their caregivers | descriptive thematic analysis |
| Challenges Faced by Family Caregivers: Multiple Perspectives on Eldercare | Strommen J, Fuller H, Sanders GF, Elliott DM. | 2020 | N = 110 | North Dakota | ND Family Caregiver Support Program (196 participants), and a Statewide Caregiving Stakeholder Survey | identify challenges to family eldercare provision from the perspectives of both caregivers and community stakeholders | thematic content analysis |
| Lay Caregivers' Experiences with Caring for Persons with Dementia: A Phenomenological Study | Mayo AM, Siegle K, Savell E, Bullock B, Preston GJ, Peavy GM. | 2020 | N = 11 | Southeastern US | semi-structured interviews in-person | describes the lived experience of caregivers caring for PWD, including their experience with palliative care | analyzed through van Manen's approach to thematic analysis |
| Resistiveness to Care as Experienced by Family Caregivers Providing Care for Someone with Dementia | Spigelmyer PC, Hupcey JE, Smith CA, Loeb SJ, Kitko L. | 2018 | N = 8 | Pennsylvania | In-person semi-structured interviews at a mutually agreed location | family caregivers lived experiences of resistiveness to care when they provided care for people with dementia | Data analysis followed the scientiﬁc phenomenological method |
| Spousal Dementia Caregiving to Widowhood: Perceptions of Older Urban and Rural Widows | Saunders MM, Groh CJ. | 2020 | N = 22 | United States | Serial, qualitative interviews were conducted at baseline, 6-months, and 12-months; in person interview in home | Clarify perceptions of widows on their transition from spousal dementia caregiving to widowhood | thematic analysis |
| Caregiving Experience of Dementia among Korean American Family Caregivers | Kim HJ, Kehoe P, Gibbs LM, Lee JA. | 2019 | N = 18 | Southern California | Semi-structured interviews in Korean or English | explored the caregiving experience of Korean American (KA) families of persons with dementia | thematic content analysis |
| "I've learned to just go with the flow": Family caregivers' strategies for managing behavioral and psychological symptoms of dementia | Polenick CA, Struble LM, Stanislawski B, Turnwald M, Broderick B, Gitlin LN, Kales HC. | 2020 | N = 26 | United States | Focus groups with 5-11 members (all caregivers) and a trained facilitator | examine family caregivers’ strategies for managing behavioral and psychological symptoms of dementia | Content analysis |
| Navigating the Complexities of Dementia Care: The Lived Experiences of Black American Family Caregivers | Ramos MD, Spearman J, Heath J, Lee D, Adewuyi M. | 2023 | N = 5 | Suburban Georgia | one-on-one interviews with systematically scripted questions | examine the experiences of Black American caregivers of individuals with ADRD | interpretive phenomenology |
| Positive Aspects of Family Caregiving for Older Adults at End-of-Life: A Qualitative Examination | Hovland CA, Mallett CA. | 2021 | N = 30 | Greater Michigan Region | qualitative, exploratory design with in-depth interviews | identifying and understanding the ongoing positive aspects of caregiving to better inform behavioral healthcare professional’s efforts and interventions | directed content analysis |
| Caregiving for Older Adults with Dementia During the Time of COVID-19: A Multi-State Exploratory Qualitative Study | Yan K, Sadler T, Brauner D, Pollack HA, Konetzka RT. | 2023 | N = 64 | Arkansas, Florida, Illinois, Minnesota, New York, North Carolina, Oregon, and Texas | qualitative semi-structured interviews | rigorously explored caregivers lived experiences and perceptions of policies that impacted their pandemic-era decision-making and wellbeing across different care settings and states that vary in HCBS utilization | thematic and content analysis, iterative coding |
| Who's Your Family?: African American Caregivers of Older Adults with Dementia | Epps F, Rose KM, Lopez RP. | 2019 | N = 26 | Southeast Louisiana | in-depth, semi-structured interviews | examine and characterize family networks of AA family caregivers who provide care to family members with dementia | inductive thematic analysis |
